# Characterization and Simulation of Shear-Induced Damage in Selective-Laser-Sintered Polyamide 12

**DOI:** 10.3390/ma17010038

**Published:** 2023-12-21

**Authors:** Daniela Schob, Lukas Richter, Krzysztof Kotecki, Dariusz Kurpisz, Robert Roszak, Philipp Maasch, Matthias Ziegenhorn

**Affiliations:** 1 Chair of Engineering Mechanics and Machine Dynamics, Faculty of Mechanical Engineering, Electrical and Energy Systems, Brandenburg University of Technology Cottbus-Senftenberg, 01968 Senftenberg, Germanymatthias.ziegenhorn@b-tu.de (M.Z.); 2 Institute of Applied Mechanics, Poznan University of Technology, 60-965 Poznan, Poland

**Keywords:** selective-laser-sintered polyamide 12 (PA12), shear loading, user material routine (UMAT), Chaboche model, GTN model, viscoplasticity

## Abstract

This paper presents the characterisation of selective-laser-sintered (SLS) samples of polyamide 12 (PA12) under shear loading. PA12 is a semi-crystalline thermoplastic and is used in various industries. Its behaviour under shear stress, which is particularly important for product reliability, has not yet been sufficiently investigated. This research focuses on understanding the material and damage behaviour of PA12 under shear-induced stress conditions. The study included quasi-static experiments and numerical simulations. Samples were prepared via SLS and tested according to ASTM standards. Digital image correlation (DIC) was used for precise deformation measurements. The Chaboche material model was used for the viscoplastic behaviour in the numerical simulations. Due to existing material discontinuities in the form of voids, the material model was coupled with the Gurson–Tvergaard–Needleman (GTN) damage model. A modified approach of the GTN model was used to account for low stress triaxiality under shear loading. These models were implemented in MATLAB and integrated into Abaqus via a User Material (UMAT) subroutine. The results of the experiments and simulations showed a high degree of accuracy. An important finding was the significant influence of the shear factor $k_{w}$ on the damage behaviour, especially during failure. This factor proved to be essential for the accurate prediction of material behaviour under shear-induced stress conditions. The integration of the modified GTN model with the Chaboche material model in UMAT enables an accurate prediction of the material and damage behaviour and thus makes an important contribution to the understanding of the mechanical material behaviour of SLS PA12 specimens.

## 1. Introduction

The advancement of technology necessitates the creation of innovative materials. Polymers are becoming increasingly important as a construction material with additive manufacturing (AM) methods being utilised. By using AM technology, a digital computer-aided design (CAD) is converted into real objects, enabling a rapid transition from design to end product. This is achieved by building up the objects layer by layer, without the need for moulding or mechanical processing [1]. During their service life, 3D-printed parts produced in this way must be able to withstand various mechanical loads. Knowing the required strength under different loads is essential [1,2]. A frequently used AM process for polymers is powder bed sintering, also known as SLS [3]. In this process, one or more lasers selectively fuse powder particles on the surface, layer by layer [4,5,6]. PA12 is one of the most commonly used materials for the SLS process. This semi-crystalline thermoplastic is ideal for functional prototypes or end-use parts due to its mechanical properties, flexibility, and heat resistance [3,7,8]. The adaptability of SLS PA12 is demonstrated by its successful integration in a wide range of industries, from the manufacture of high-performance ball valves and medical devices to customised automotive parts and beyond [3,9,10,11,12,13]. In order to utilise the full potential of SLS PA12, a thorough understanding of the material properties under a wide range of loading conditions is essential. In recent years, studies have been conducted on the material behaviour of SLS PA12 under both quasi-static and cyclic loading [14,15,16,17,18]. Due to the lack of comprehensive standards in the field of AM, the procurement and interpretation of material data remain difficult [19]. One particular area to be investigated is the behaviour of SLS PA12 under shear-induced stress conditions, an area that is relatively under-researched [20,21,22]. The challenge is not only to identify the details of the material behaviour, but also to determine the most suitable test geometries that can provide reliable and reproducible results, especially in shear-dominated scenarios [23,24,25,26]. Even though the current state of the art in SLS is advanced, it also has its limitations. The process can lead to structural inhomogeneities in the final product in the form of voids, microcracks, or even unsintered areas [16,27,28,29,30]. These microstructural features in combination with the described complex material properties pose challenges for numerical modelling. In the work of [15,16], the material model of Chaboche [31] was coupled with the damage model of Gurson–Tvergaard–Needleman [32], and a high accuracy between experiment and simulation was achieved. The GTN model does not take shear stress into account. In this respect, attempts have been made in recent years to modify this model, following the work of Xue [33] and Nahshon and Hutchinson [34], who presented a framework that potentially provides higher accuracy under combined tensile and shear loading.

The present work has three objectives. The first one is the experimental characterisation of the material and damage behaviour of SLS PA12 under shear load. The second aim of the study is to couple Chaboche’s material model with the modified GTN damage model based on Nahshon and Hutschinson’s approach to ensure that it captures the details of shear-induced behaviour with optimal precision. Due to the limited data available for SLS PA12 in common FE software (ABAQUS 2022) material libraries, the third objective of this work is to implement the material and damage model in a UMAT.

## 2. Experiments

### 2.1. Sample Preparation

The specimens were fabricated utilizing SLS technique. A PA2200 powder was deployed for the process. A sPro 230 3D printer, manufactured by 3D Systems and functioning at a power output of 70 W, was utilized in the fabrication. The printer operated at a laser scanning speed of 10 m/s for the infill and 5 m/s for the contour delineation. The nominal layer thickness was maintained in the range of 0.08 to 0.15 mm. The process and the chamber temperature during the print cycle were sustained at 200 °C and 170 °C, respectively.

**Figure 1:**
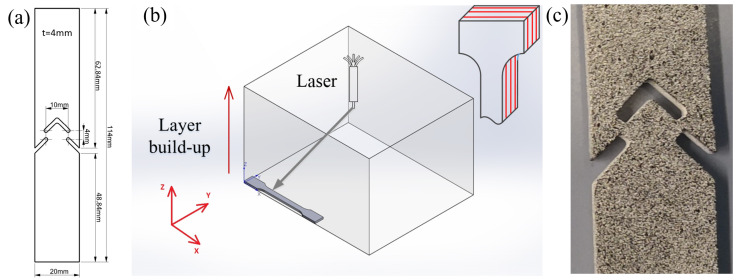
ASTM shear specimen: (**a**) geometry, (**b**) single printing layers are in the xy-plane, the build-up direction of the layers is the z-direction; and (**c**) black and white pattern for DIC.

Given the current lack of standardized testing procedures for AM polymers under shear loading [19], the testing geometry was chosen in line with the ASTM B831 standard [35], as illustrated in Figure 1a. Based on prior research, it has been observed that the resultant material properties demonstrate only minimal dependence on the printing direction [15]. Therefore, all subsequent tests were performed on specimens layered along the *z*-axis, Figure 1b.

### 2.2. Digital Image Correlation

**Figure 2:**
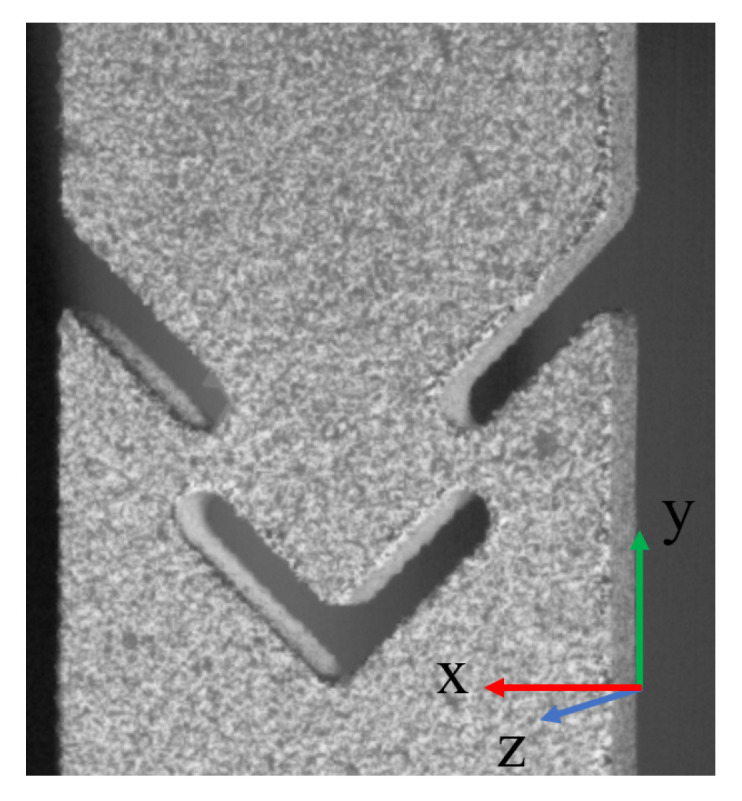
Reference image of DIC in Aramis.

For the execution of DIC, the ARAMIS 3D Camera measurement system was deployed for comprehensive area-based and discrete point measurements. DIC is an optical non-contact method, allowing for full-field measurement of deformations. The technique relies on a reference image to serve as a baseline for deformation measurements. Consequently, a unique binary (black and white) pattern is applied to the specimen surface (Figure 1c). These stochastic patterns facilitate the identification of discrete image areas, with subpixel accuracy, through the analysis of image-based information, Figure 2.

The methodology generates point and area measurement results which are subsequently used in material research and component testing to evaluate the static behaviour of specimens [36]. The fundamental correlation function is represented as [37]:

(1) $$C\left(x,y,u,v\right)=\sum_{i,j=-N/2}^{N/2}{\left(I\left(x+i,y+j\right)-I^{*}\left(x+u+i,y+v+j\right)\right)}^{2}$$

where *C* is a function of the coordinates *x* and *y* of the reference image. The displacement and disparity are defined as *u* and *v*, respectively. *I* represents the pre-deformation image and is a function of the pixel values x+i and y+j. The post-correlation, $I^{*}$, signifies the image function of the pixel values after the application of displacements.

The function C(x,y,u,v) represents the cumulative squared differences between the reference and the deformed images, where the function is summed over the subset size *N*.

Using the Hegewald & Peschke Inspekt-Table 10 kN testing machine (Nossen, Germany), five shear tests were conducted at a 1 mm/min displacement rate and a temperature of 23 °C.

## 3. Numerical Simulation

### 3.1. Chaboche Model

Previous investigations have shown that SLS PA12 behaves viscoplastically [15]. This study focuses on the development of material-specific adjustments of the Chaboche material model, with limitations set on purely isothermal conditions. The Chaboche model is constituted of a system of differential equations, originating from the division of the total strain rate ${\dot{\varepsilon }}^{tot}$ into an elastic ${\dot{\varepsilon }}^{el}$ and a viscoplastic ${\dot{\varepsilon }}^{vp}$ component [38]:

(2) $${\dot{\varepsilon }}^{tot}={\dot{\varepsilon }}^{el}+{\dot{\varepsilon }}^{vp},$$

(3) $$\dot{\sigma }=E\;:\left({\dot{\varepsilon }}^{tot}-{\dot{\varepsilon }}^{vp}\right),$$

with the stress σ and Young’s Modulus E. Herein, the definition of the viscoplastic strain rate ${\dot{\varepsilon }}^{vp}$, composed of the viscoplastic multiplier $\dot{\lambda }$ and the direction ∂f/∂σ, is decisive:

(4) $${\dot{\varepsilon }}^{vp}=\dot{\lambda }\frac{\partial f}{\partial \sigma }={\left(\frac{f}{K}\right)}^{n}\left(\frac{\partial f}{\partial \beta^{v}}\frac{\partial \beta^{v}}{\partial \sigma }+\frac{\partial f}{\partial \beta^{H}}\frac{\partial \beta^{H}}{\partial \sigma }\right).$$

The index (·)${}^{v}$ indicates that it is an equivalent stress and (·)${}^{H}$ hydrostatic stress. The relative stress β is defined by [39]

(5) $$\beta =\sigma -\left(1-\hat{f}\right)\sum_{k=1}^{2}X^{k},\;\;\mathrm{with}\;k=1,2.$$

The flow function *f* delineates the closure of the elastic domain and $X^{k}$ the non-linear kinematic hardening. Equation (6) defines the flow function of viscoplastic behaviour [32]:

(6) $$f={\left(\frac{\beta^{v}}{\sigma^{M}}\right)}^{2}-1+2q_{1}{\hat{f}}^{*}cosh\left(\frac{3q_{2}}{2}\frac{\beta^{H}}{\sigma^{M}}\right)-{\left(q_{1}{\hat{f}}^{*}\right)}^{2}\geq 0.$$

The parameters $q_{1}$ and $q_{2}$ are damage parameters and ${\hat{f}}^{*}$ is the void volume fraction. The flow function is dependent on the non-linear isotropic hardening $\sigma^{M}$ [38]:

(7) $$\sigma^{M}=\sigma^{y}+Q^{\infty }\left(1-exp\left(-b{\bar{\varepsilon }}^{vp}\right)\right)+K{\left({\dot{\bar{\varepsilon }}}^{\;vp}\right)}^{1/n},$$

with the accumulated plastic strain rate ${\bar{\varepsilon }}^{vp}$, where its derivative is the modulus of plastic strain rate:

(8) $${\dot{\bar{\varepsilon }}}^{vp}=\frac{\sigma \;:\;{\dot{\varepsilon }}^{vp}}{\left(1-{\hat{f}}^{*}\right)\sigma^{M}}.$$

The isotropic hardening $\sigma^{M}$ describes the extension of the flow surface and is defined by the yield stress $\sigma^{y}$, the viscosity factor *K*, the viscosity exponent *n*, and the hardening parameters $Q^{\infty }$ and *b*. In addition to the isotropic hardening, the flow function is influenced by the non-linear kinematic hardening X. The kinematic hardening depends on the strain hardening parameter *C*, the dynamic recovery factor γ, the static recovery factor $R^{kin}$, the decay constant ω, and the saturation parameter $\varphi_{\infty }$. The first term in Equation (9) describes the strain hardening and the second and third ones describe the dynamic and static recovery, respectively [40]:

(9) $$\dot{X}=\sum_{i=1}^{2}\left(\frac{2}{3}C^{\left(i\right)}{\dot{\varepsilon }}^{vp}-\gamma^{\left(i\right)}\varphi^{\left(i\right)}X^{\left(i\right)}{\dot{\bar{\varepsilon }}}^{vp}-R^{kin(i)}X^{\left(i\right)}\right),$$

with:

(10) $$\varphi =\sum_{i=1}^{2}\left(\varphi_{\infty }^{\left(i\right)}+\left(1-\varphi_{\infty }^{\left(i\right)}\right)\exp\left(-\omega^{\left(i\right)}{\bar{\varepsilon }}^{vp}\right)\right).$$

### 3.2. GTN Damage Model

The results of the micromechanical analysis from a previous investigation [15] were taken into account for the selection of the damage model. The virgin samples have a large portion of nearly spherical voids. Due to the quasi-static and cyclic loading, the voids are growing. When the spaces between the voids become small enough coalescence occurs. This coalescence needs to be embedded in the GTN model [32] so that it takes into account pore growth, nucleation, and coalescence.

In Equation (11), the effective void volume fraction $f^{*}$ is presented. This models the correlation between the reduction in the material’s load-bearing ability and coalescence [41].

(11) $${\hat{f}}^{*}=\left\{\begin{array}{cc} {\hat{f}}_{0} & \mathrm{if}\;t_{0}=0\\ \hat{f} & \mathrm{if}\;\hat{f}\leq {\hat{f}}^{c}\\ {\hat{f}}^{c}+\frac{\frac{1}{q_{1}}-{\hat{f}}^{c}}{{\hat{f}}^{F}-{\hat{f}}^{c}}\left(\hat{f}-{\hat{f}}^{c}\right) & \mathrm{if}\;{\hat{f}}^{c}<\hat{f}\leq {\hat{f}}^{F}. \end{array}\right.$$

The initial porosity, ${\hat{f}}_{0}$, is set at t=0. As the void volume approaches the critical limit ${\hat{f}}^{c}$, coalescence begins. This continues until the material reaches the failure void volume fraction ${\hat{f}}^{F}$ and subsequently fails. To describe the void volume fraction $\hat{f}$, modifications were made to the original GTN model. Nahshon and Hutchinson’s [34] approach was utilized, which considers the impact of shear load on damage behaviour. As a result, the shear term ${\hat{f}}_{Shear}$ was added [34]:

(12) $$\hat{f}={\hat{f}}_{0}+\left({\dot{\hat{f}}}_{Shear}+{\dot{\hat{f}}}_{Growth}+{\dot{\hat{f}}}_{Nucleation}\right)dt,$$

so the void volume fraction $\hat{f}$, is characterized by the sum of the initial void volume ${\hat{f}}_{0}$, void growth ${\hat{f}}_{Growth}$, void nucleation ${\hat{f}}_{nucleation}$, and void volume fraction due to shear ${\hat{f}}_{Shear}$. The increment in the void volume fraction due to pore growth ${\dot{\hat{f}}}_{Growth}$ is described by the effective void volume fraction ${\hat{f}}^{*}$ and the viscoplastic strain rate ${\dot{\varepsilon }}^{vp}$ [32]:

(13) $${\dot{\hat{f}}}_{Growth}=\left(1-{\hat{f}}^{*}\right)Sp\left({\dot{\varepsilon }}^{vp}\right).$$

Assuming a strain-controlled load, the nucleation of pores follows a normal Gaussian distribution. This has a mean value $\varepsilon^{N}$ and a standard deviation $S^{N}$. The new pore volume created via nucleation corresponds to the parameter ${\hat{f}}^{N}$. The nucleation is strongly dependent on the matrix material [32]:

(14) $${\dot{\hat{f}}}_{Nucleation}=\frac{{\hat{f}}^{N}{\dot{\bar{\varepsilon }}}^{vp}}{S^{N}\sqrt{2\pi }}exp\left(-\frac{1}{2}{\left(\frac{{\bar{\varepsilon }}^{vp}-\varepsilon^{N}}{S^{N}}\right)}^{2}\right).$$

Nahshon and Hutchinson formulated an equation to predict the increase in void volume fraction at low stress triaxiality, expressed as follows [34]:

(15) $${\hat{f}}_{shear}=k_{w}\frac{{\hat{f}}^{*}\cdot w\left(\sigma \right)}{\sigma^{v}}S\;:\;{\dot{\varepsilon }}^{vp},$$

with the shear factor $k_{\omega }$, the deviatoric stress tensor S, and the stress state function [34],

(16) $$w\left(\sigma \right)=1-{\left(\frac{27J_{3}}{2{\left(\sigma^{v}\right)}^{3}}\right)}^{2},$$

with the third invariant of the deviatoric stress tensor $J_{3}=\left|S\right|$.

All the described parameters of Equations (2)–(16) were adjusted using the pattern search algorithm [42] and subsequently incorporated into Abaqus for the execution of the numerical simulation.

### 3.3. UMAT in Abaqus

Initially, the coupled Chaboche-GTN model was implemented in Matlab for one point. The return mapping algorithm is an iterative method used in plasticity theory [43,44]. For each incremental step of the simulation, an elastic prediction is made first. Then, it is checked whether the resulting stress meets the yield condition. If the yield condition has been reached, it is assumed that a plastic deformation has occurred and a correction must be made. This correction, namely the determination of the corresponding plastic deformation increment to “return” the stress to the yield surface, is performed by the return mapping algorithm [43].

The model parameters of the coupled Chaboche-GTN model were determined using a pattern search algorithm [42] in MATLAB together with the experimental data from the shear tests. This process ensured that the simulation results accurately reflected the actual experimental results. The main goal of applying the pattern search algorithm was to fit the model as closely as possible to the actual physical phenomena in order to improve its accuracy and reliability. This step is crucial for validating the effectiveness of the model and its ability to accurately replicate real physical processes. Table 1 lists the parameters determined. The modulus of elasticity *E*, the yield stress $\sigma^{y}$, the initial void volume fraction ${\hat{f}}_{0}$, and the void volume fraction at failure ${\hat{f}}^{F}$ were determined directly from the experiments. All other parameters were determined by optimisation.

**Figure 3:**
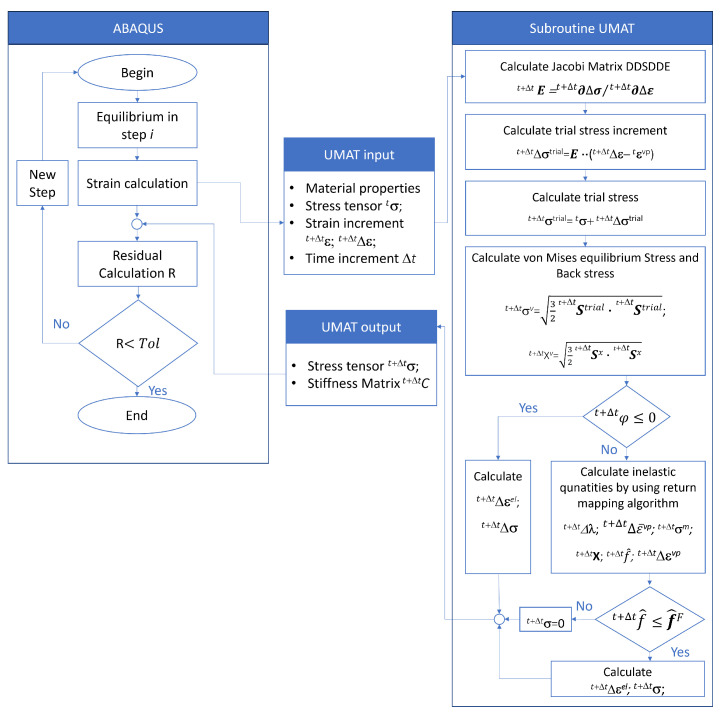
UMAT scheme.

For the integration of the model into the finite element analysis software Abaqus 2022, the UMAT function was utilized. UMAT is a subroutine within Abaqus 2022 that allows users to define and implement custom material models in FORTRAN. This function provides the flexibility to model complex material behaviours that go beyond the models typically provided in Abaqus. A schematic overview of the process flow and the implementation of the applied model is depicted in Figure 3. The parameters derived from Matlab were used as input variables for the finite element analysis.

**Figure 4:**
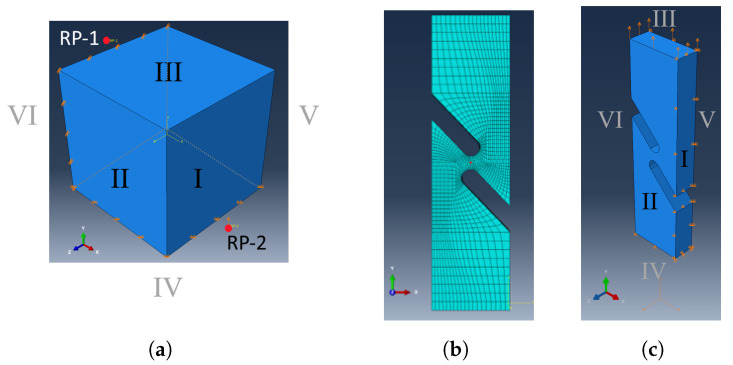
(**a**) Boundary condition simple shear model (**b**), mesh of half model, (**c**) boundary condition of the half model.

For the finite element simulation, a reference model for the “simple shear” load case was created to cross-check the results of the UMAT in MATLAB. The C3D8 element was chosen for this purpose, a three-dimensional element with eight nodes and a linear shape function. With the support of constraints and the “Equations” function, these points were linked to the respective surfaces. Specifically, RP-1 was associated with the displayed surface III in the x-direction, and RP-2 in the y-direction for surface I. In the load case, the movement of RP-1 and its associated surface was fixed in the x-direction, while RP-2 underwent a defined displacement in the y-direction. For surface VI, the displacement in both the z and y directions was set to zero. Similarly, for surface IV, the displacement in the x and z directions was also set to zero. To simulate “simple shear”, two reference points, RP-1 and RP-2, were introduced, as shown in Figure 4a.

In addition to the reference model, a half-model of the original geometry was used, as shown in Figure 1. This half-model is depicted in Figure 4b. The boundary conditions are defined as follows: The displacement is constrained in the x-direction on Surface I, in the y-direction on Surface IV, and in the z-direction on Surface V, Figure 4c. A displacement in the y-direction is prescribed on Surface III. For this model, the C3D8 element type was taken.

## 4. Results and Discussion

### 4.1. Experimental Results

**Figure 5:**
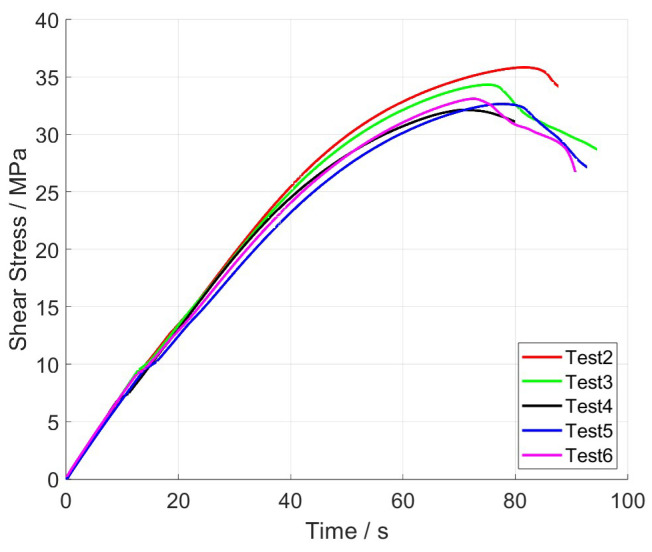
Experimental results of shear stress over time.

The results of the five experiments are visualised in the shear stress–time diagram in Figure 5.

The mean value $\bar{x}$ of the shear modulus and ultimate strength determined from these five shear tests and the corresponding standard deviations *s* are listed in detail in Table 2.

(17) $$\bar{x}=\frac{1}{n}\sum_{i=1}^{n}x_{i}\;s=\sqrt{\frac{1}{n}\sum_{i=1}^{n}\left(x_{i}-\bar{x}\right)}$$

The standard deviation values, 10.34 MPa for shear modulus and 2.51 MPa for ultimate strength, suggest a consistent performance of the material across different samples. Test 4 was used for the further fitting of the model parameters.

### 4.2. Simulation Results

**Figure 6:**
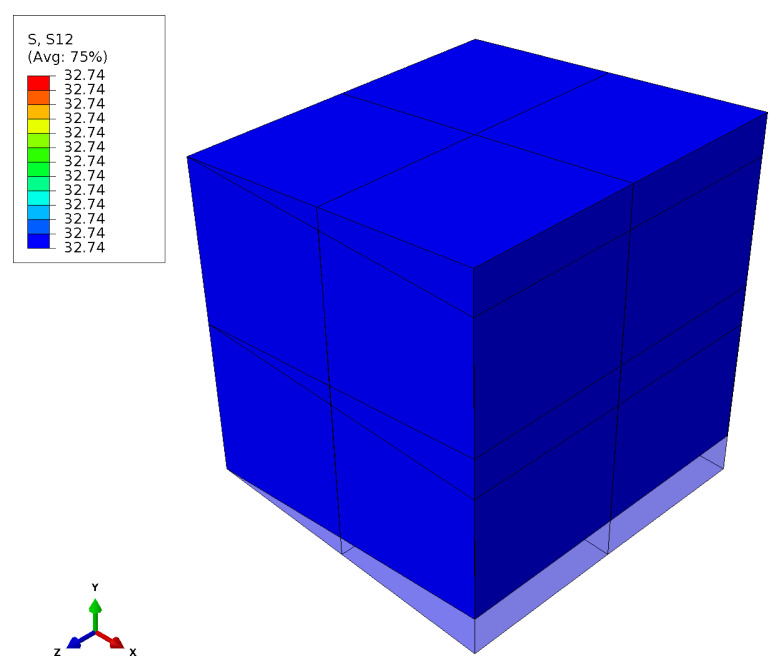
Shear stress.

Prior to the analysis, a thorough verification process was conducted to ensure the integrity of the transferred code from MATLAB to UMAT. After reviewing the model in MATLAB and integrating it into the UMAT, the FE model for simple shear (Figure 4) was analysed. In Figure 6, the deformed body and the associated shear stress $\sigma_{12}$ are depicted.

**Figure 7:**
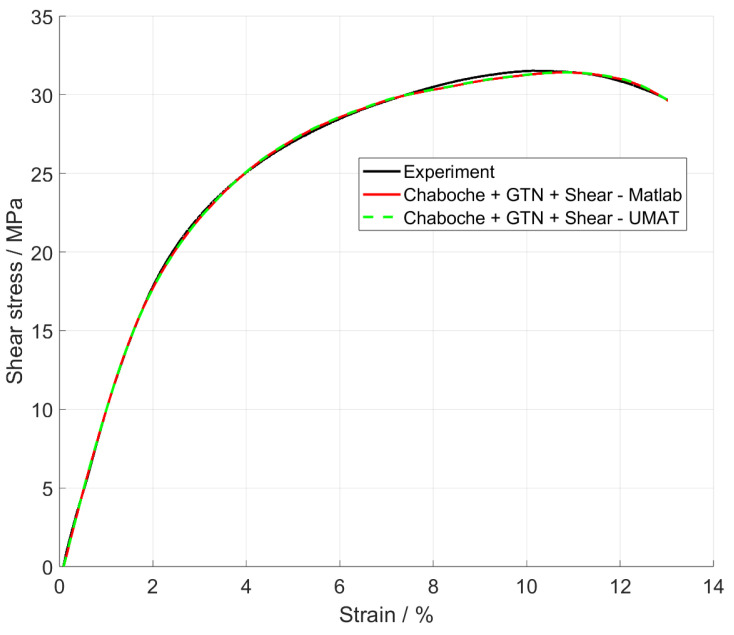
Shear stress over strain.

A strong correlation was observed between the results from MATLAB (red line) and the UMAT (green dotted line) in comparison to the experimental data (black line), Figure 7. Both the material behaviour and the time of failure could be simulated exactly.

**Figure 8:**
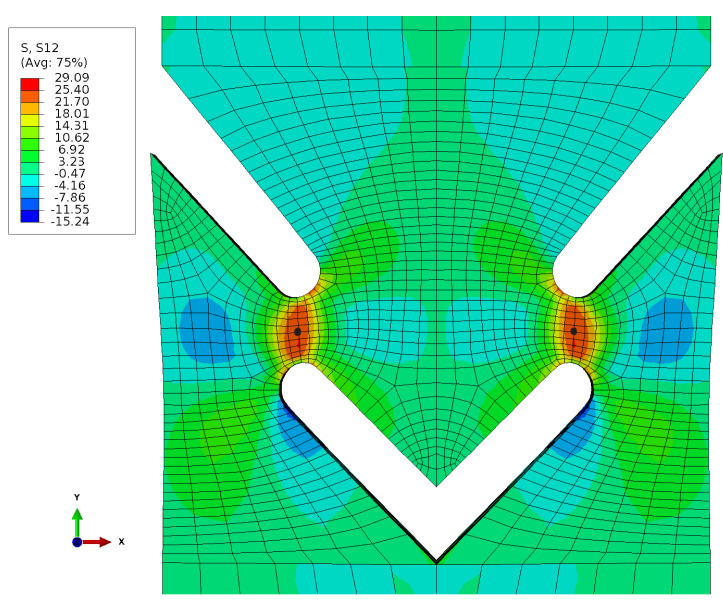
Stress field $\sigma_{12}$.

As expected, an increased shear stress $\sigma_{12}$ was observed in the region of the webs (Figure 8).

**Figure 9:**
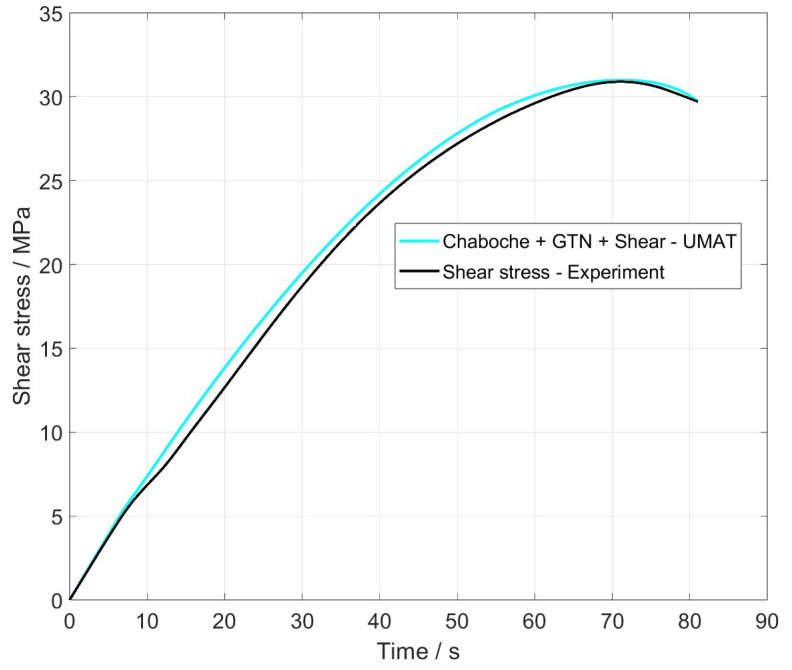
Shear stress over time of the node of full model.

The analysis of the shear stress $\sigma_{12}$ at a node (black point) in the shear zone and its comparison to the experiment are illustrated in Figure 9. The material and damage behaviour can be mapped with a very high accuracy. The maximum deviation is 1 MPa and occurs at the point in time when the shear and tensile loads superimpose each other.

**Figure 10:**
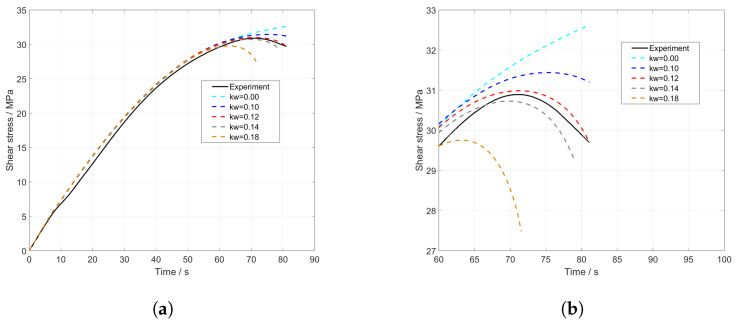
(**a**) Shear stress full model of UMAT and (**b**) cutout of stress–time curve under the influence of $k_{w}$.

Furthermore, the influence of the newly introduced shear factor $k_{w}$ (Equation (15)) was examined (Figure 10a). The value for $k_{w}$ determined through optimization is 0.12 (Figure 10a, dotted red line). To analyse its influence, this value was increased to 0.18 and decreased to 0. The results are depicted in the stress–time diagram, both throughout the entire course (Figure 10a) and in detail shortly before failure (Figure 10b).

From Figure 10b, it becomes evident that $k_{w}$ has a significant influence on the characterization of the damage behaviour, especially shortly before failure. When the value of $k_{w}$ decreased, the stress is overestimated, while it is underestimated when increased. Misestimating this value can lead to inaccurate stress and failure predictions. Therefore, it is essential to calibrate $k_{w}$ correctly to ensure that model predictions align with the real damage behaviour.

**Figure 11:**
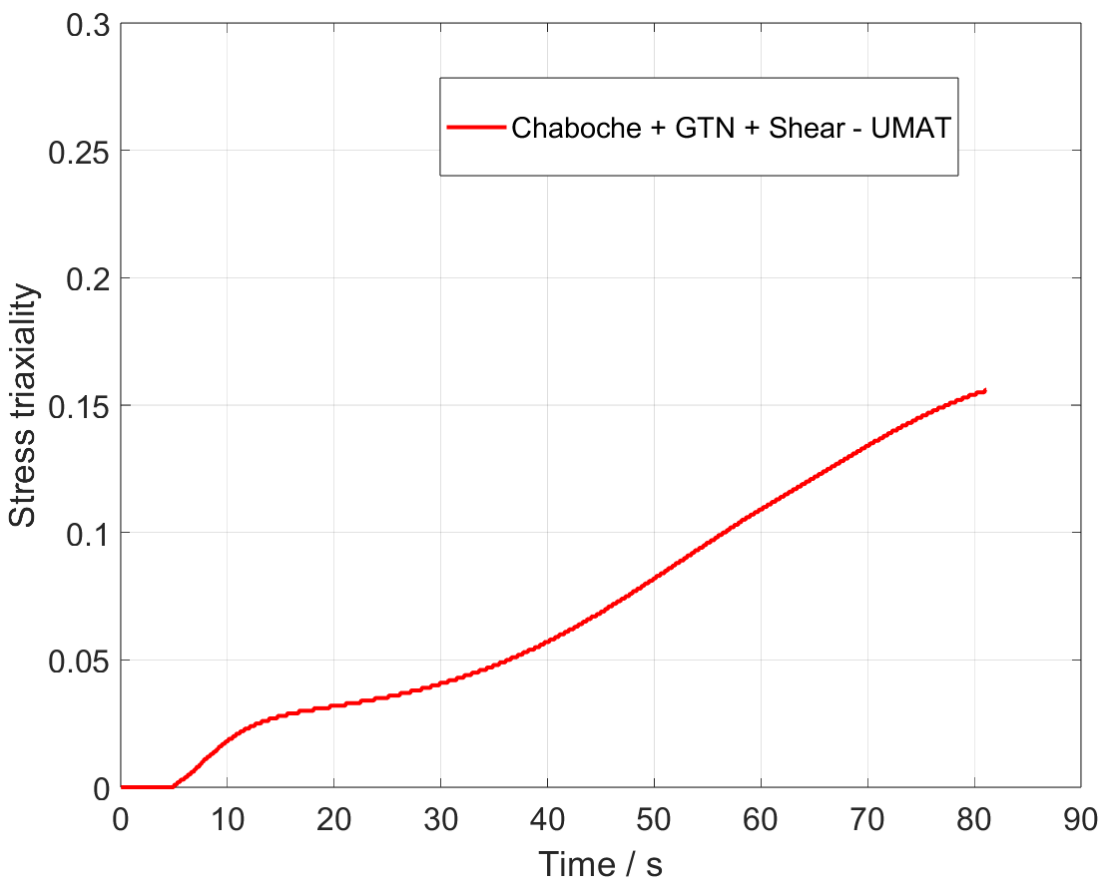
Stress triaxiality of full model of UMAT.

In order to investigate whether pure shear or a superposition of shear and tension is present in the component, the calculation of the stress triaxiality η,

(18) $$\eta =\frac{-\sigma^{H}}{\sigma^{v}},$$

provides a statement [45,46]. The stress triaxiality indicates the ratio of hydrostatic stress $\sigma^{H}$ to the Mises equivalent stress $\sigma^{v}$. A high value of the stress triaxiality η=0.33 indicates that pure tensile or compressive stress predominates. Low values indicate pure shear stress. Figure 11 illustrates the stress triaxiality in the same node as the analysis of the shear stress (Figure 8). At the beginning of the loading, there is pure shear stress. As the load progresses, the stress triaxiality increases to a value of η=0.17 and a combination of shear and tensile stresses occurs, with shear stress remaining the predominant factor. The results presented in Figure 9 demonstrate that the enhanced GTN model is capable of accurately representing both pure shear stresses and the combined loading scenario of shear and tension.

**Figure 12:**
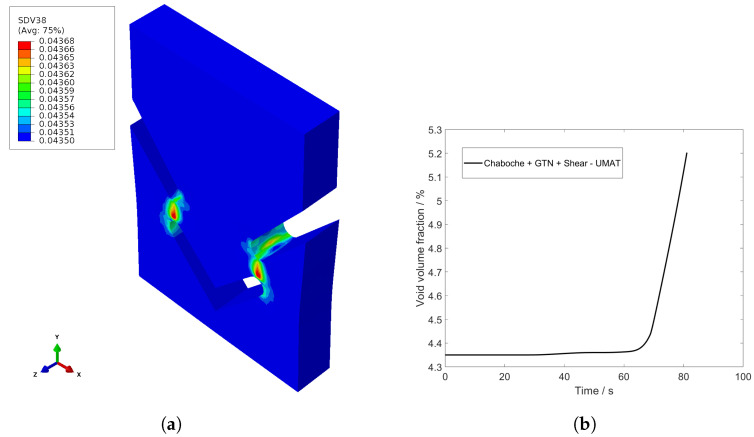
(**a**) Void volume fraction of full model of UMAT, t=40 s and (**b**) development of void volume fraction over time.

The programmed material and damage model in UMAT allows for the representation of individual development variables. In Figure 12, the void volume fraction at the time t=40 s is depicted as ${\hat{f}}^{*}$. The complete progression of the void volume fraction over time is shown in Figure 12. Until the yield point is reached, the void volume fraction retains its initial value ${\hat{f}}_{0}$. As soon as the yield point is exceeded, the void volume fraction gradually increases. When the coalescence threshold ${\hat{f}}_{c}$ is reached, the increase in the void volume fraction accelerates significantly.

**Figure 13:**
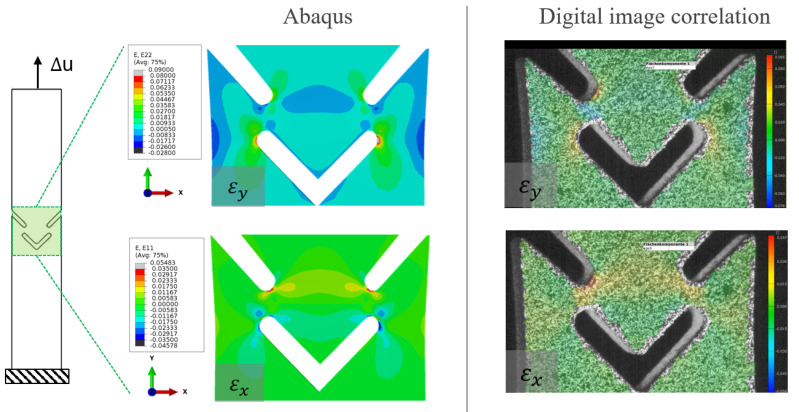
Comparison of the strain field of Abaqus and DIC in y-direction and x-direction.

In Figure 13, the results from Abaqus and the DIC are compared. The focus is on the comparison of the deformation fields, especially the strains in the x and y directions. The evaluation is conducted for t=40 s. An excellent agreement between the experiment and the simulation is evident.

## 5. Conclusions

This study investigated the material and damage behaviour of SLS PA12 under shear loading and the application of Chaboche’s material model with the modified GTN model. The main conclusions are the following:

1. This study provides fundamental insights into the behaviour of SLS PA12 under shear loading, which are relevant for a wide range of applications in materials science and 3D printing.
2. The application of the Chaboche material model in combination with the modified GTN model shows that complex material behaviours, such as those of SLS PA12 under shear loading, can be successfully simulated, improving the prediction accuracy in similar material studies.
3. The results of this study extend the understanding of the damage behaviour of 3D-printed materials and provide a valuable contribution to the further development of reliable simulation models.

In summary, the precise determination of the shear factor $k_{w}$ enables an accurate simulation of the material and damage behaviour of SLS PA12, which opens perspectives for improved design and testing methods in additive manufacturing.

## Figures and Tables

**Table 1:** Determined parameters.

Parameter	Unit	Value
Poisson ratio ν		4.13
Young’s modulus *E*	MPa	1203.00
Yield stress $\sigma^{y}$	MPa	8.00
Viscosity factor *K*	MPa	11
Viscosity exponent *n*		2.7
Hardening parameter $C^{1}$	MPa	593.7
Hardening parameter $C^{2}$	MPa	457.97
Recovery factor $\gamma^{1}$		138.97
Recovery factor $\gamma^{2}$		126.50
Hardening parameter $Q^{\infty }$		126.50
Hardening parameter *b*		28.65
Static recovery factor $R^{kin(1)}$		0
Static recovery factor $R^{kin(2)}$		0
Saturation parameter $\varphi_{\infty }^{1}$		0
Saturation parameter $\varphi_{\infty }^{2}$		0
Decay constant $\omega^{1}$		0
Decay constant $\omega^{1}$		0
Initial void volume fraction ${\hat{f}}_{0}$		0.0435
Damage parameter $q_{1}$		0.7
Damage parameter $q_{2}$		0.5
Failure void volume fraction ${\hat{f}}^{F}$		0.052
Nucleation void volume fraction ${\hat{f}}^{N}$		1.4 × 10${}^{-4}$
Mean value $\varepsilon^{N}$		0.0173
Standard deviation $S^{N}$		0.1536
Void volume fraction coalescence ${\hat{f}}^{C}$		0.0438
Shear factor $k_{w}$		0.12

**Table 2:** Mean values and standard deviation.

	Unit	Mean Value	Standard Deviation
Shear modulus	MPa	667.37	10.34
Ultimate strength	MPa	32.22	2.51

## Data Availability

The raw/processed data required to reproduce these findings can be shared with interested researchers upon request.
